# Editorial Chit-Chat

**Published:** 1876-07

**Authors:** 


### Editorial Chit-Chat.

FLORIDA LETTER.—Of course our readers will turn to the pleasant letter of Dr. Gleason's, on Florida. The doctor always sees with both eyes wide open, and has a droll way of telling us what those eyes of his have been looking at.

THE FLORIDIANS.—Quite a large number of our citizens migrated to Florida, last winter, and almost every one of them have been ill, since their return, with malarial fever. They have abandoned oranges and alligators, and have taken to quinine, for a steady diet.

FIVE-YEAR-OLDS.— As pretty a sight as can well be seen, is to look in upon forty or fifty five-year-old children, dressed in their best, and engaged in celebrating the birth-day of one of their number. If you have a five-year-old, make him or her a birth-day party. It's fun!

H, AT LAST.—Since H's return from Florida, he has been discussing the fly and flea difficulty pretty thoroughly, but whether he has determined which is wisest, to fly to a flea, or to flee from a fly, does not clearly appear in the article in this number of the BISTOURY. Our friends must read and decide for themselves.

TRICHINA.—The people of Savona, in this state, have had a great fright over several deaths that have occurred in their town from trichina-disease. All the cases of sickness were traced to one porker, whose flesh was found to be alive with *trichina-spiralis.* Old father Moses was right when he declared the hog unclean and unfit for food. Confound the stuff,—cook it thoroughly, so as to kill, if possible, all the trichina, then throw it out at the window.

PHOTOGRAPHING EXTRAORDINARY.—Van Aken, than whom a better photographic artist our city never saw, recently had a call from a gentleman who was very anxious to procure a picture of his wife. "Certainly," said the artist, "bring her in." "Ah!" replied the customer, "there's the trouble, she's dead." "Then you have a picture you desire copied," replied the artist. "Not at all," says the customer, "that is just what I'm after—I have no picture of her and I want you to make one while I tell you how she looks!"

Mr. Van Aken not being in the "spiritual photograph" business, of course, lost a customer.

MR. TOWNER'S POEM.—The State Editorial Association made a capital selection of their poet this year, in Mr. Ausburn Towner, city editor of the *Advertiser.* Mr. Towner has made a wide reputation as an able writer, so that the honor conferred by the state society of Editors, naturally fell to him. His poem, of course, is the very best that has yet appeared in the proceedings of this association, and does great credit to our talented townsman.

LEFT OVER.—We have prepared an illustrated article upon *trichina spiralis,* which we expected to have printed in this number of our journal, but the crowded condition of the several departments, has lessened our editorial space, and crowds it out for the present. In the meantime, we can assure our readers all the same, that pork is not fit to eat—that nearly all of it is diseased, and that much sickness and death is the direct cause of its use. Leave off your pork-and-beans, ham-and-eggs, and employ a more wholesome and less dangerous food.

CAMPING OUT.—By the time this number of the BISTOURY reaches our readers, the editor will have thrown down his pencil, and with his old friend H., may be found on the Lycoming, snugly quartered in a comfortable tent, surrounded by hills, streams and flowers. And, if you could find us, dear reader, we could exhibit to your vision as beautiful a creel of trout as ever was caught by happy fishermen, and perchance cook them for you too. So bye, bye, until October, with its beautiful foliage and charming scenery, brings us face to face again.

COMPROMISES WITH NATURE.—Our readers have long before now remarked the excellence of the articles furnished this journal by our faithful contributor Mr. Ben. H. Pratt. From the very beginning of the BISTOURY, Mr. Pratt has never failed to furnish our readers with a capitally entertaining and well written paper, many of which we have found extensively copied in various magazines and journals throughout the land, so attesting to their popularity and usefulness. We say it modestly, and believe it sincerely, that no better magazine articles appear anywhere than those furnished the BISTOURY by Mr. Pratt. The present one, is in his happiest vein, and will doubtless receive a careful reading from all his friends.

Mr. Pratt is the City Editor of the Scranton, (Pa.) *Times*, and as our readers will readily believe, furnishes a witty and exceedingly spicy journal.

OUR DRIVING PARK.—It is becoming generally known that Elmira has one of the best and *the* best managed driving park in the country. The association have erected magnificent buildings, have built a capital track, offer princely premiums, and conduct the whole affair in a decorous and strictly unobjectionable manner, so that their entertainments are patronized by ladies and children as well as by gentlemen. The Driving Park Association is a credit and real benefit to our city. May they continue always to receive the success which they so richly merit.

———

FLOWERS.—No sort of open air exercise is provocative of better and more satisfactory results than the cultivation of flowers. You not only obtain exercise, but constant enjoyment in inspecting the results of your labor, for nothing can be more beautiful than a dooryard full of flowers. We invested a few dollars in Mr. Vick's seeds, constructed a hot bed for their germination, prepared ornamental beds for the reception of the plants, and now rejoice in such a floral display as cannot be easily excelled. For real healthful, outdoor pastime, nothing can surpass the cultivation of flowers. Try it!

———

THE CENTENNIAL.—The exhibition at Philadelphia in honor of our hundredth year of national existence, has proved to be a grand success in every particular. The exhibition in industrial articles, from all parts of the world, is the finest and largest that has ever been collected. Aside from the exorbitant charges that are made by most of the Philadelphia hotels, nothing remains of which any fault can be found. The managers of the exhibition have been most liberal in supplying all manner of conveniencies for visitors and are very moderate in their charge. The grand rush of visitors will occur in the fall months, for which almost every one seems to be waiting. We hope our southern friends will come up and visit with us, when we will give them a hearty, cheerful welcome.

———

"THRUSH."—This is the name of a horse disease, or rather a disease of a horse's hoof. In our particular case, it is a disease of our mare's hoof, which we suppose is quite as bad. Now, diseases of horses are queer—queer in that they have so many remedies. We have been recommended to apply twenty-eight separate and distinct medicines for "thrush," by as many different "horse-men." The antagonism of many of these is somewhat peculiar. We have been asked to burn the hoof with a red-hot poker. The next chap recommended ice to the parts. Cleaning it thoroughly with soap and water, and then—daubing it with tar. Filling it full of salt—then, cauterizing it with nitrate of silver. Standing her feet on moist ground—place her on hard, dry plank. Place her in the shade—put her in the sun. Turn her into a box-stall—tie her fast in an open stall. We have tried to avoid all these kind recommendations, and have treated poor Fanny, with her sore foot, much upon the same plan that we would treat a human being's "hoof," and are glad to note that she is doing well.

———

FAITHFUL OFFICIALS.—Such an individual is rarely to be found, at this day. Elmira, bountiful in her supply of good things, can also furnish more than her quota of good and true men,—men noted for their honesty, business capacity, and trustworthiness. We accidently overheard a gentleman of this character tell a friend that he had been in the employ of the Erie Railway for *twenty-three years*, and, that during that time he had lost but *seven days* from duty. This is the record of our respected and beloved fellow citizen, Mr. Chauncey W. Gardner, Superintendent of Susquehanna Division of the Erie Railway.

———

AMERICAN GIRL STATUE.—The statue erected to the memory of the famous trotter "American Girl," who fell dead on the Elmira Driving Park track, one year ago, is a beautiful work of art, certifying to the excellent and refined taste of the gentlemen who compose that association. The statue is life size, beautifully and artistically modeled, while it is said to be a faithful likeness of the animal it commemorates. It stands upon a large block of granite, which in turn rests upon a grassy mound which is shaded by a well proportioned oak tree. In front, is an urn of beautiful flowers, the whole forming a very beautiful picture, well worth a visitation to the park to see. The munificence of Dr. Eldridge, who has furnished us with the beautiful Eldridge Park, with its flowers and statuary, and now the good taste of the gentlemen of the Driving Park, who have likewise beautified their grounds, renders our city quite enjoyable for lovers of the beautiful in Art and Nature.

———

VALUABLE CROSS.—A knight of the rod, in this city, related the following incident to us today. In a recent fishing excursion, composed of several gentlemen, one of whom was a clergyman, when within a short distance of a noted resting place, called "Brown's," within nine miles of Glenn's Falls, a resort, famous for its milk punches, it was proposed to the clergyman that they stop and partake of Brown's milk, and as an inducement, expatiated much upon the peculiar qualities of Brown's lacteal fluid,—the delicacy of its flavor, and the richness of its "boquet." Finally, Brown's was reached, when one of the party slyly ordered the usual milk punches, which they offered the minister as a sample of the milk referred to. Of course, it was much enjoyed—the clergyman smacking his lips thereon, and praising, with unusual fervency and zeal the wonderful merits of the milk. In the midst of one of his eloquent apostrophes to milk, the landlord appeared, when the clerical fisherman ventured to enquire: "Landlord, what sort of a cow is it, that gives this excellent milk?" "She is a cross between the Alderney and Santa Cruz," sir, was the prompt reply of mine host, and our clerical friend was satisfied, and ordered another glass.

---

PLUMBER'S HELP.—He's a queer chap. Ever watch him? It is worth the three dollars a day you will be charged for his services (?) just to take him in. While the plumber is scraping a quantity of black paint from the end of a pipe that he has just taken the greatest of pains to place there, in the most artistic manner,—measured by the time he "kills" in doing it, ye helper seeks your easiest chair, rubs the smut from his overalls thereon, and with a squirt of tobacco juice, aimed at your fire-place, but which he signally fails to hit, he proceeds to peruse your morning paper and discusses with you the prospects of Conkling or Tilden for the next presidency. Reluctantly, we have come to the conclusion that the plumber's "helper" is a brilliant piece of unnecessary, and I may add, impudent, extravagance; while as a conversationalist, or *companion du voyage, etc.*, why, he's simply a miserable failure.

---

BUILDERS' ESTIMATES.—Ever build a barn? A house, perhaps? No? It's fun! It's funny in many ways. First, to look on, and take a hand at it yourself, with hammer and nails, one-half the time picking up nails and the other half removing hemlock slivers from your hands and other exposed portions of your body. Funny is it, too, to see what becomes of all that pile of hemlock 2x4's, 3x6's, 2x6's, and hear the men talk about joist, studs, rafters, sills, ribbons, stay-lath and sundry other technicalities. Then, its funny, after your barn is done, to find enough lumber left to build another, when you sit down and wonder whether you will open a lumber yard on your own hook, or build another barn just to save the confounded stuff. But funnier still is it to pay the bill. Aye, there's the rub.

We have learned, to build a barn, don't make a balloon frame—too many 2x6's, and all that sort of thing, in it. Make your estimate as to what it will cost, then add one-third for "contingencies," about a sixteenth for oddities, one-quarter for idiosyncrasies of builder, one-half for your own nonsense, then multiply the result by four. In this manner you will be able to approximately guess at the cost of that barn.

---

IT MAKES A DIFFERENCE.—Once, when paddling through one of the mountain streams of Pennsylvania, amusing ourself in witnessing the leaps of the sprightly trout as they left the beautitul crystal water to grasp the artificial fly which we were playing above their heads, our meditations as well as our sport was startlingly interrupted by a messenger, who pushed his way through the deep underbrush until he found himself within hailing distance, when: "Hellow! stranger, be you the Doctor?" was his greeting. We replied that we were *a* doctor, when he announced that a telegram had come for us at the station, and that "some of your folks have been run over by the cars, and they want you to hum!" Well, we got to that station, but *how*, will ever remain a mystery to us. After we learned that it was an unfortunate employee of the road, and not one of our own little ones that had suffered such a terrible injury, we found that we had succeeded in getting out of the woods with our paraphernalia all in good order, but we do not want an experience of that sort again. If ever a poor fellow's foot was amputated carefully, tenderly and *thoughtfully*, it was done upon that particular day. We have found it to make a deal of difference *whose* foot you are amputating—always sad for any one, terribly so, it must be, in your own family. So our fright taught us to think.

---

There is great interest at present manifested in the question whether the Exhibition grounds and buildings shall be opened upon Sunday. The Catholics and a majority of foreigners favor the opening. The proprietors of liquor-selling establishments are especially favorable to such an action. Several mass-meetings have been held with a view to influence the commissioners to open the grounds, at least, if not the buildings. It is still uncertain what will be the final decision.—*Phila. Ledger.*

